# Identifying Contact Time Required for Secondary Transmission of *Clostridioides difficile* Infections by Using Real-Time Locating System

**DOI:** 10.3201/eid3005.231588

**Published:** 2024-05

**Authors:** Min Hyung Kim, Jaewoong Kim, Heejin Ra, Sooyeon Jeong, Yoon Soo Park, Dongju Won, Hyukmin Lee, Heejung Kim

**Affiliations:** Hallym University Dongtan Sacred Heart Hospital, Hwasieong, South Korea (M.H. Kim);; Yonsei University College of Medicine, Seoul, South Korea (J. Kim, D. Won, H. Lee);; Yonsei University Yongin Severance Hospital, Yongin, South Korea (H. Ra, S. Jeong, Y.S. Park, H. Kim)

**Keywords:** *Clostridioides difficile*, *Clostridioides difficile* infection, horizontal transmission of infectious diseases, secondary transmission, hospital-acquired infections, patient isolation, real-time locating system, bacteria, enteric infections, South Korea

## Abstract

Considering patient room shortages and prevalence of other communicable diseases, reassessing the isolation of patients with *Clostridioides difficile* infection (CDI) is imperative. We conducted a retrospective study to investigate the secondary CDI transmission rate in a hospital in South Korea, where patients with CDI were not isolated. Using data from a real-time locating system and electronic medical records, we investigated patients who had both direct and indirect contact with CDI index patients. The primary outcome was secondary CDI transmission, identified by whole-genome sequencing. Among 909 direct and 2,711 indirect contact cases, 2 instances of secondary transmission were observed (2 [0.05%] of 3,620 cases), 1 transmission via direct contact and 1 via environmental sources. A low level of direct contact (113 minutes) was required for secondary CDI transmission. Our findings support the adoption of exhaustive standard preventive measures, including environmental decontamination, rather than contact isolation of CDI patients in nonoutbreak settings.

Mitigating the incidence of *Clostridioides difficile* infections (CDIs), particularly those acquired in healthcare settings, has received increased attention because of the notable prevalence of this contagion (*1*,*2*). Although the incidence of hospital-acquired CDI has declined because effective infection control measures have been used (*3*), the effectiveness of specific interventions used to curb disease transmission remains unclear (*4*–*6*). The efficacy of contact isolation for symptomatic patients has been questioned because recent reports have highlighted the transmission of *C. difficile* by asymptomatic carriers (*4*). Considering patient room shortages in resource-limited settings and the endemicity of other pathogens, such as carbapenemase-producing Enterobacterales and coronaviruses causing COVID-19 (*7*,*8*), isolating symptomatic patients with CDI requires revaluation. Acquiring data on the secondary transmission rate of CDI is crucial and should emphasize comprehensive patient contact histories, regardless of specific points of contact.

Real-time locating system (RTLS) technology is well suited for acquiring data on secondary CDI transmission rates; the system can be leveraged to precisely quantify human-to-human interactions irrespective of the number of contacts (*9*–*11*). RTLS involves radio-frequency identification and a wireless network tracking system, which calculates the distance and duration of human-to-human interaction by analyzing the signal from a radio-frequency identification tag worn by users (*12*). Although concerns regarding privacy and cost–benefit persist, accumulating evidence supports the validity of using RTLS technology in hospital settings (*9*,*10*,*13*).

Since its inception, Yongin Severance Hospital in South Korea has been equipped with RTLS, which can provide epidemiologic data for patient contact time and distance with high sensitivity. We aimed to determine the real-world CDI transmission rate by using RTLS, focusing on the contact time required for infection transmission in susceptible patients within this hospital.

## Methods

### Ethics Statement

This study was approved by the Institutional Review Board of the Yonsei University Health System Clinical Trial Centre, and the study protocol adhered to the tenets of the Declaration of Helsinki (approval no. 9–2022–0209; approved on February 24, 2023). Because this was a retrospective study, the Institutional Review Board waived the requirement for written informed consent from the study participants.

### Study Design and Participants

We conducted a retrospective cohort study involving hospitalized patients who had direct or indirect contact with index patients who had a CDI diagnosis during September–December 2021. The study concluded on July 29, 2022, when information from the last enrolled patient was acquired. CDI was diagnosed by using PCR, which detected the *C. difficile* toxin B gene, and by identifying *C. difficile* in fecal culture samples obtained from patients experiencing diarrhea (Appendix 1). Diarrhea was defined as new-onset bowel movements >3 times per day. Yongin Severance Hospital is a university-affiliated hospital that has 560 beds; 46.7% (86/184) of rooms have 4–5 beds. Patients in the same room shared toilets, except in the intensive care unit, where most patient beds were isolated, eliminating the need for shared toilets. After discharge, the rooms were cleaned with nonsporicidal disinfectants. Although patients with CDI were not placed under specific contact isolation, the hospital used enhanced standard infection control measures throughout the hospital because of the COVID-19 pandemic, which included encouraging regular handwashing with soap and water and mask use. CDI index patients were not isolated as a contact precaution during hospitalization; their baseline characteristics were recorded (Appendix 1 Table 1).

**Table 1:** Baseline characteristics of patients in study identifying contact time required for secondary transmission of *Clostridioides difficile* infections by using real-time locating system in South Korea*

Characteristics	All patients	Patients with subsequent CDI			Patients without subsequent CDI	p value‡
		Secondary transmission	Nonsecondary transmission	p value†		
Total no. patients	2,520	2	56	NA	2,462	NA
Mean age, y (SD)	60.4 (19.8)	81.5 (2.1)	73.4 (11.6)	<0.001	60.06 (19.80)	0.091
Sex						
M	1,343 (53.3)	2 (100.0)	31 (55.4)	>0.99	1,310 (53.2)	NA
F	1,177 (46.7)	NA	NA	NA	NA	NA
Prior hospitalization	381 (15.1)	0	18 (32.1)	>0.99	263 (14.7)	0.153
Recent antimicrobials	1,686 (66.9)	2 (100.0)	54 (96.4)	>0.99	1,630 (66.2)	0.047
Underlying conditions						
Diabetes mellitus	765 (30.4)	1 (50.0)	25 (44.6)	0.881	739 (30.0)	0.600
COPD	91 (3.6)	0	6 (10.7)	>0.99	85 (3.5)	0.926
Chronic heart failure	617 (24.5)	1 (50.0)	28 (50.0)	>0.99	588 (23.9)	0.876
Hypertension	1,131 (44.9)	1 (50.0)	36 (64.3)	0.683	1,094 (44.4)	0.521
Chronic kidney disease	341 (13.5)	0	18 (32.1)	>0.99	323 (13.1)	0.633
Malignancy	640 (25.4)	1 (50.0)	21 (37.5)	0.723	618 (25.1)	0.905
IBD	1 (0.0)	0	0	NA	1 (0.0)	NA
CVA	279 (11.1)	0	11 (19.6)	>0.99	268 (10.9)	NA
HSCT	104 (4.1)	0	8 (14.3)	>0.99	96 (3.9)	0.821
Median CCI score (IQR)	2 (0–3)	2.5 (2.25–2.75)	4 (2–6)	0.447	2 (0–4)	0.387
No. days before index patient treatment, median (IQR)	1 (0–2)	1.5 (1.25–1.75)	1 (0–2)	>0.99	1 (0–2)	NA
Laboratory tests						
Blood leukocyte count >15,000/µL	244 (11.2)	0	12 (22.2)	>0.99	232 (10.9)	0.710
Median CRP, mg/L (IQR)	3.9 (0.6–34.7)	53.3 (44–62.6)	42.9 (16.3–93.2)	0.794	4.8 (0.7–40.4)	0.209
Mean albumin, g/dL (SD)	3.05 (0.54)	3.15 (0.35)	3.10 (0.55)	0.890	2.96 (0.55)	NA
Clinical conditions						
Ileostomy	29 (1.2)	0	1 (1.8)	>0.99	28 (1.1)	NA
Enteral tube insertion	325 (12.9)	2 (100.0)	21 (37.5)	>0.99	302 (12.3)	0.201
No. contact cases§	3,620	2	124		3,494	
Group 1¶	909 (25.1)	1 (50.0)	43 (34.7)	>0.99	865 (24.7)	0.516
Room sharing#	181 (19.9)	0	11 (25.6)	>0.99	170 (19.7)	NA
Contact during diarrhea episode**	316 (34.8)	0	13 (30.2)	>0.99	303 (35.0)	NA
Group 2††	421 (11.6)	0	11 (8.9)	>0.99	410 (11.7)	NA
Group 3‡‡	2,290 (63.3)	1 (50.0)	70 (56.5)	>0.99	2,219 (63.5)	NA
Median contact time, min (IQR)	4,320 (131.5–8,640)	7,976.5 (4,044.75–11,908.25)	4,320 (133.25–10,080.0)	0.465	4,320 (132–8,640)	NA
No. deaths	162 (6.4)	0	11 (19.6)	>0.99	151 (6.1)	NA

*Values are no. (%) except as indicated. CCI, Charlson comorbidity index; CDI, *Clostridioides difficile* infection; COPD, chronic obstructive pulmonary disease; CRP, C-reactive protein; CVA, cerebrovascular accident; HSCT, hematopoietic stem cell transplantation; IBD, inflammatory bowel disease; IQR, interquartile range; NA, not applicable. †A univariate logistic regression was used to compute p values, comparing secondary transmission with nonsecondary transmission. ‡A generalized linear mixed model was used to compute p values after adjusting for variables exhibiting statistical significance in the univariate analysis; the model was used to elucidate the odds of subsequent CDI occurrence. The odds ratios and 95% CIs for each variable are shown in Appendix 1 Table 2. §Because 744 patients experienced >2 episodes of contact with separate index patients, a disparity emerged between the number of contact cases and the number of contact patients. ¶Group 1 included patients who had direct contact with index patients. #Co-hospitalization in the same bedroom with the index patient for >24 hours. **Contact with index patient who had diarrhea. ††Group 2 included patients who had indirect contact with index patients via healthcare personnel. ‡‡Group 3 included patients who had indirect contact with index patients via the environment.

We tracked CDI contact cases by using 3 different methods. First, we investigated patients who came in direct contact with CDI index patients by using RTLS. We considered patients within a 1-meter radius of index patients to have had direct contact, regardless of the duration. Second, we collected data for patients who came in indirect contact with CDI index patients via healthcare personnel. We used RTLS to identify contact cases where patients interacted with healthcare personnel who had attended to an index patient for >24 hours. We assumed the disease could be potentially transmitted through healthcare workers’ hands or through fomites, such as blood pressure cuffs. We systematically calculated contact duration for the entire hospitalization period, irrespective of the presumed contagiousness of the index patient. We adopted this approach to ensure the comprehensive inclusion of patients susceptible to transmission during the asymptomatic phase of the index patient. Third, we identified CDI cases arising from indirect contact through environmental contaminants. We enrolled patients who were hospitalized in the same rooms as index patients within 3 months after the index patient’s discharge. The patients were followed up until their last outpatient visit or hospitalization. We tracked diarrhea symptoms and obtained results for *C. difficile* toxin B gene PCR and for the fecal *C. difficile* culture tests from electronic medical records.

The primary outcome was secondary CDI transmission, identified by whole-genome sequencing. We performed PCR ribotyping for all *C. difficile* isolates obtained from the patients with a CDI diagnosis. Among the designated contacts, we sequenced whole genomes of paired *C. difficile* samples from patients harboring identical ribotypes. We determined person-to-person transmission by examining the genetic relatedness of isolates to reveal consistent core genome sequence types and substantial allelic homogeneity. We excluded index patients with a history of CDI within 3 months before the study period, contact case-patients with a history of diarrhea but without laboratory tests to confirm CDI, and contact case-patients who had a short follow-up period of <7 days.

### PCR Ribotyping

We performed PCR ribotyping by using the primers CD1–CD1445 (*14**,**15*). We compared PCR ribotyping patterns with those of known standard *C. difficile* strains (VPI10463, UK078, 48489ATCC9689, ATCC43598, and ATCC70057). We considered ribotype patterns with >1 band difference to be different ribotypes.

### Whole-Genome Sequencing

We generated sequencing libraries for *C. difficile* genomic DNA by using Twist Library Preparation EF 2.0 Kit and Twist UDI Primers (Twist Bioscience, https://www.twistbioscience.com) according to the manufacturer’s instructions. We extracted genomic DNA by using the chemagic 360 extraction instrument and chemagic DNA Tissue Kit (both PerkinElmer, https://www.perkinelmer.com). We assessed the quantity of DNA in the libraries by using Qubit 3.0 and the Qubit dsDNA HS Assay Kit (ThermoFisher Scientific, https://www.thermofisher.com) and assessed quality by using the 4200 TapeStation and DNA1000 ScreenTape (Agilent, https://www.agilent.com). We used the quantified final library products for cluster generation and performed next-generation sequencing on an Illumina NovaSeq 6000 sequencer system (Illumina, https://www.illumina.com) in 300-bp paired-end format according to the Illumina paired-end sequencing protocol. We performed de novo assembly of sequences by using Unicycler version 0.4.8 (https://github.com/rrwick/Unicycler) and analyzed core genomic multilocus sequence typing by using EnteroBase (https://enterobase.warwick.ac.uk).

Among isolate pairs with the same ribotype, 2 pairs of identical core genomic sequence types had allelic differences of 9 and 13. We distinguished between secondary and nonsecondary transmission according to the distribution of allelic differences among pairs of identical ribotypes (104 [interquartile range 27–1,709] differences). The probability of genetic homogeneity was statistically significant for the same core genomic sequence types with allelic differences <13 (p = 0.010).

### Contact Tracing with Real-Time Locating System

The hospital used RTLS sensors designed to detect signals within a 2-meter radius in bedrooms and within a 10-meter radius in open spaces throughout the facility. Hospital staff and inpatients were required to always wear the RTLS tags. The tags emitted signals every 1–3 seconds, confirming the presence of the person in a specific room. The distance between persons was calculated through a tag-to-tag signal interaction. When 2 persons were at a particular distance from each other, the contact time between them was counted, enabling data collection for the cumulative contact time between the 2 persons. 

### Statistical Analysis

We used a generalized linear mixed model and a logit link function to model CDI occurrence. The fixed effects in the model encompassed various factors, including age, prior hospitalization, recent antimicrobial use, the elapsed time before treatment of the CDI index patient, comorbidities (diabetes mellitus, chronic obstructive pulmonary disease, congestive heart failure, hypertension, chronic kidney disease, malignancy, inflammatory bowel disease, cerebrovascular accident, and hematopoietic stem cell transplantation), Charlson comorbidity index scores, categorized leukocyte counts, serum levels of C-reactive protein and albumin, presence of ileostomy, insertion of enteral tube, and contact type. In addition, the model incorporated random intercepts for time and an unstructured covariance matrix. For the generalized linear mixed model, only variables demonstrating an effect on CDI occurrence were selected as fixed effects from baseline data. We conducted a univariate logistic regression to determine the influence of each variable on secondary transmission within the group that developed subsequent CDI. For analysis of categorical variables, we used frequencies and percentages for descriptions; for continuous variables, we used means and SDs. We performed statistical analyses and created graphs by using both SPSS Statistics 26.0 (IBM Corp, https://www.ibm.com) and R version 4.2.2 (The R Project for Statistical Computing, https://www.r-project.org). We conducted all statistical tests with a significance level set at 0.05.

## Results

### Patient Characteristics

Adherence to wearing the RTLS tags was 91.3% (interquartile range 90.5%–92.6%) during the study. We identified 4,196 contact cases for 26 index patients, of which 490 were excluded because of short follow-up periods and 86 were excluded because of a lack of laboratory results, despite a history of diarrhea. A disparity emerged between the number of contact cases and number of contact patients because 744 contact patients experienced >2 episodes of contact with separate index patients. Consequently, we defined instances of contact as contact cases and persons who experienced contact episodes as patients. Among the remaining 3,620 contact cases (comprising 2,520 patients), 2,587 (71.5%) cases were followed up for >30 days. The number of contact cases attributed to direct contact was 909/3,620 (25.1%); 2,711 contact cases resulted from indirect contact occurring either through healthcare personnel (421/3,620 [11.6%]) or through environmental exposure (2,290/3,620 [63.3%]) (Appendix 1 Figure 1). Within the subset of 909 direct contact cases, 181 (19.9%) instances involved patients who shared a bedroom with an index patient for >24 hours; 728 (80.1%) contact cases involved diverse encounters, such as during radiologic exams, rehabilitation, physiotherapy, or brief encounters occurring within the confines of the same bedroom. Furthermore, 316 (34.8%) direct contact cases were identified when the index patients exhibited symptoms of diarrhea, whereas 593 (65.2%) contact cases were identified during an index patient’s asymptomatic phase (Table 1).

The mean age (+SD) of the 2,520 contact patients was 60.37 (+19.76) years; 53.3% (1,343) were men and 46.7% (1,177) women. We identified a history of hospitalization in 15.1% and recent antimicrobial use in 66.9% of all contact patients. Among contact patients, 4.1% (104) received hematopoietic stem cell transplantation, whereas 25.4% (640) had a history of malignancies. Only 1 patient with a history of inflammatory bowel disease was included in the study. All index patients underwent treatment for CDI, which was initiated ≈1 day after identifying the infection. The median contact time was 4,320 (interquartile range 131.5–8,640) minutes. Among the 2,520 patients that had follow-up, CDI was diagnosed in 58 patients. Recent antimicrobial use was greater (96.4%) for patients with a subsequent CDI diagnosis than for those without a subsequent CDI diagnosis (66.2%; p = 0.047) (Table 1; Appendix 1 Table 2). We identified ribotypes of *C. difficile* isolates from index patients and from contact patients who had a subsequent CDI diagnosis (Appendix 1 Figure 2). Ribotype 014/016 had the highest (23.1%) prevalence, whereas ribotype 018 had a lower (8.9%) prevalence than previously described (*16*).

### Identifying Secondary Transmission of *C*. *difficile* Infection

Of 126 contact cases involving 58 patients with a subsequent CDI diagnosis, 13 contact cases (11 patients) had the same *C. difficile* ribotype as their index patient. Two patients had secondary transmission of *C. difficile*; each was associated with a distinct index patient. One secondary transmission occurred through direct contact, whereas the other occurred via exposure to environmental sources (2 of 3,620 cases; 0.05% incidence rate). The mean age of patients with secondary transmission (81.50 +2.12 years) was greater than that of patients with nonsecondary transmission (73.38 +11.58 years; p<0.001) (Table 1).

The patient who had secondary *C. difficile* transmission through direct contact with an index patient did not cohabit in the same room. The contact duration was 113 minutes and occurred during the asymptomatic phase of the index patient. The patient with indirect environmental contact was hospitalized 36 days after discharge of the index patient; the contact time was 11 days (Table 2). Neither patient had a hospitalization history; however, they both had a history of recent antimicrobial use and insertion of an enteral tube. Ribotype 018 was associated with both instances of secondary transmission (Tables 1, 2). We defined the secondary transmission rate as the ratio of the cumulative number of secondary transmissions to the total number of contact cases per unit of contact time (Appendix 1 Figure 3). The rapid decrease in transmission rate after the initial surge (1 of 948 cases; 0.001% at 113 minutes), followed by a plateau was attributed to the brief contact time necessary for secondary transmission (Appendix 1 Figure 3).

**Table 2:** Patients manifesting secondary transmission in study identifying contact time required for secondary transmission of *Clostridioides difficile* infections by using real-time locating system conducted in South Korea*

Patient age, y	CCI score	Reason for hospitalization	Ribotype	Contact during diarrhea episode†	Contact time, min	Contact type‡	Indwelling devices
81	3	Pneumonia	RT018	No	113	Group 1	Enteral tube, pleural effusion drainage
83	2	Pneumonia	RT018	NA	15,840	Group 3	Enteral tube

*CCI, Charlson comorbidity index; NA, not applicable; RT018, ribotype 018. †Contact history during manifestation of diarrhea in the index patient. ‡Group 1 included patients who had direct contact with index patients. Group 3 included patients who had indirect contact with index patients via the environment.

## Discussion

Our findings demonstrate a low contribution of patient contact to CDI transmission. However, we found that a low level of direct contact time was required for secondary transmission of CDI. In-hospital transmission rates observed in previous studies have varied according to the surveillance methods used (*17*–*19*). Most studies have focused on finding the sources of hospital-acquired CDI, which has led to analyses of only confirmed cases, and susceptible patients at risk of contracting the infection have not been extensively evaluated. A precise rate estimation can be made by using the correct choice of susceptible patients in the denominator. In this study, the transmission rate estimations were made by using RTLS. The comprehensive detection capability of RTLS in contact tracing was exemplified by the substantial percentage of contact cases identified beyond shared bedrooms (Table 1). The overall CDI transmission rate (0.05%) observed in this study was lower than that identified in a prospective study conducted at a tertiary hospital in Switzerland (*17*). That study used stringent standard precautions instead of patient isolation, and the subsequent secondary transmission was investigated among patients who had contact with CDI patients. The number of secondary transmission cases in that study, even without including cases of asymptomatic transmission, was comparable to the number in our study. Nevertheless, RTLS identified both direct and indirect contact cases, which have been previously overlooked. In addition, contact cases in our study were distinguished from contact patients; some patients had multiple episodes of contact, mirroring real-world dynamics.

The duration of person-to-person contact required for CDI transmission in our study was as brief as 113 minutes. Infection dose of *C. difficile* is known to be low in a laboratory setting, but those results have not yet been supported in vivo (*20*,*21*). This study investigated the association between contact time and secondary transmission of CDI. A low contact time required for CDI transmission might help explain the absence of differences in CDI incidence rates for genetically related and genetically distant strains, despite the use of contact precautions, as previously described (*18*). Short infection periods for multiple *C. difficile* spreaders have been reported, emphasizing that organism density is more crucial for transmitting the disease than longer contact time (*22*). Patients can spread spores, which can be taken up by susceptible patients within hours, depending on organism density. Therefore, once a patient starts showing symptoms, intervention would be considered delayed. Furthermore, multiple CDI cases identified in this study were categorized as asymptomatic transmission, which is a subject of concern (*4*,*6*,*23*). Because of adherence to augmented standard precautions in our hospital throughout the study period and considering the role of indirect contact through environmental CDI transmission (*24*), it might be more pragmatic to adopt exhaustive standard preventive measures rather than opting for contact isolation of symptomatic patients with CDI. A comprehensive strategy should encompass additional preventive measures, such as careful excrement management and environmental decontamination.

The overall incidence of CDI in the study institution was ≈19.6 cases/10,000 patient-days in 2021, signifying a notable increase compared with 5.9 cases/10,000 patient-days reported in tertiary hospitals within South Korea during 2020–2021 (*25*). This study was conducted in an environment marked by substantial transitions from long-term care facilities, resulting in a high incidence of imported cases, which contributed to the elevated overall incidence rate. Despite the high CDI incidence in this study compared with previous research, the effect of secondary transmission via direct or indirect contact on CDI incidence was found to be low. Consequently, factors contributing to disease occurrence that are distinct from CDI patient contact warrant investigation. Previous studies have highlighted the significance of prudent antimicrobial use to diminish spontaneous sporulation of toxigenic *C. difficile* (*26*–*30*). Therefore, this precautionary measure should be prioritized, particularly in a setting where a high percentage of patients might experience dysbiosis because of immobility.

The first limitation of our study is that we could have underestimated the secondary transmission rate by not accounting for asymptomatic carriers who could potentially harbor *Clostridioides* spores. However, the optimal timing for collecting rectal swab samples to detect secondary transmission in low-risk patients remains uncertain (*31*). Therefore, the best approach for ascertaining the secondary transmission rate involves estimation of identified symptomatic patients. Second, RTLS serves as a surrogate metric for contact identification; however, RTLS performance evaluation was precluded in this study because of challenges in identifying a suitable counterpart. Nevertheless, RTLS is characterized by its high sensitivity (*32*) and proves advantageous for investigating CDIs when the mode of transmission remains incompletely elucidated (*5*,*18*). Our findings retain importance by revealing only 2 instances of secondary transmission after a comprehensive investigation. Third, this study was conducted in an environment where highly contagious strains, such as ribotype 027 and ribotype 018, were infrequently identified. Of note, both instances of secondary transmission observed in this study were linked to ribotype 018, which is well known for its multidrug resistance and transmission capabilities (*33*,*34*). We acknowledge that different study outcomes might vary according to the predominant ribotypes, emphasizing the importance of incorporating ribotyping results in outbreak investigations. Fourth, the timely identification of CDI cases by following hospital policy and immediate treatment of CDI-confirmed patients could have contributed to the low transmission incidence observed in this study. We recommend exercising caution in extrapolating our results to other environments.

In conclusion, our study showed a low incidence of secondary CDI transmission within a short period of direct contact. Thus, our findings support prioritizing the comprehensive use of standard preventive measures in healthcare facilities, including environmental decontamination, as a more viable approach to prevent *C. difficile* infection than relying on symptom-based contact isolation of patients in nonoutbreak settings.

Appendix 1 Additional information for identifying contact time required for secondary transmission of *Clostridioides difficile* infections by using real-time locating system.

Appendix 2 Raw data used for identifying contact time required for secondary transmission of *Clostridioides difficile* infections by using real-time locating system.
