# Supplementary material for: Identifying Contact Time Required for Secondary Transmission of Clostridioides difficile Infections by Using Real-Time Locating System
Source: Emerg Infect Dis. 2024 May;30(5):908–15. doi: 10.3201/eid3005.231588 (PMC11060456; doi:10.3201/eid3005.231588)
Supplement: Appendix 1 — Additional information for identifying contact time required for secondary transmission of Clostridioides difficile infections by using real-time locating system. [file 23-1588-Techapp-s1.pdf]

Article DOI: https://doi.org/10.3201/eid3005.231588

EID cannot ensure accessibility for supplementary materials supplied by authors. Readers who have difficulty accessing supplementary content should contact the authors for assistance.

Identifying Contact Time Required for Secondary Transmission of *Clostridioides difficile* Infections by Using Real-Time Locating System

Appendix 1

Additional Methods

Isolation of *Clostridioides difficile*

A standardized method was used to identify *C. difficile*. Fecal samples were subjected to alcohol shock and subsequently cultured anaerobically on CHROM CDIF agar (Asanpharm, https://www.asanpharm.com) for 48 h at 35 ±2°C. The bacteria were identified by using a Bruker Biotyper matrix-assisted laser desorption/ionization–time-of-flight mass spectrometer (Bruker Daltonics, https://www.bruker.com).

PCR

Real-time PCR was performed to detect the toxin B gene in fecal specimens. The presence of the toxin B gene was determined by using the Xpert *C. difficile* assay (Cepheid, https://www.cepheid.com) according to the manufacturer's instructions.

Appendix 1 Table 1. Baseline characteristics of index patients in a study investigating secondary transmission rate of *Clostridioides difficile* infection through quantified measurements via a real-time locating system, South Korea*

Characteristics	Index patients, n = 26
Mean age, y (SD)	74.46 (11.35)
Sex	
M	14 (53.8)
F	12 (46.2)
Prior hospitalization	2 (7.7)
Recent antimicrobials	26 (100.0)
Comorbidities	
Diabetes mellitus	13 (50.0)
COPD	2 (7.7)
Chronic heart failure	11 (42.3)
Hypertension	14 (53.8)
Chronic kidney disease	5 (19.2)
Malignancy	1 (34.6)
IBD	0 (0.0)
Cerebrovascular accident	5 (19.2)
HSCT	0 (0.0)
Median CCI score (IQR)	3 (1.5–4)
Ribotypes	
RT014/020	6 (23.1)
RT018	5 (19.2)
Clinical information	
Presence of diarrhea	26 (100.0)
Severe CDI†	9 (34.6)
Fever	19 (73.1)
Ileostomy	0 (0.0)
Enteral tube insertion	9 (34.6)
No. treated for CDI	26 (100.0)
Median hospital stay, d (IQR)	20 (7.5–33.5)

*Values are no. (%) except as indicated. CCI, Charlson comorbidity index; CDI, *Clostridioides difficile* infection; COPD, chronic obstructive pulmonary disease; HSCT, hematopoietic stem cell transplantation; IBD, inflammatory bowel disease; IQR, interquartile range; RT, ribotype.

†Severe CDI was diagnosed when the blood leukocyte count was >15,000/mm^3, serum creatinine level was ≥1.5 times higher than the level before illness, or when the patient suffered from shock.

Appendix 1 Table 2. Univariate and multivariable analysis of subsequent *Clostridioides difficile* infection risk in study investigating secondary transmission rate of CDI through quantified measurements by using a real-time locating system in South Korea*

Patient characteristics	Patients with subsequent CDI, n = 58	Univariate analysis†		Multivariable analysis‡	
		OR (95% CI)	p value	aOR (95% CI)	p value
Mean age, y (SD)	74.50 (11.03)	1.049 (1.033–1.066)	<0.001	1.008 (0.999–1.018)	0.091
Sex					
M	33 (56.9)	0.860 (0.508–1.454)	0.573	NA	NA
F	25 (43.1)	NA	NA	NA	NA
Prior hospitalization	18 (31.0)	2.617 (1.484–4.616)	0.001	1.426 (0.877–2.318)	0.153
Recent antimicrobials	56 (96.5)	14.336 (3.490–58.892)	<0.001	1.478 (1.006–2.173)	0.047
Comorbidities					
DM	26 (44.8)	1.900 (1.125–3.211)	0.016	0.747 (0.252–2.218)	0.600
COPD	6 (10.3)	3.216 (1.344–7.694)	0.009	0.955 (0.365–2.503)	0.926
CHF	29 (50.0)	3.188 (1.889–5.378)	<0.001	1.053 (0.548–2.023)	0.876
HTN	37 (63.8)	2.198 (1.279–3.776)	0.004	0.820 (0.447–1.503)	0.521
CKD	18 (31.0)	2.969 (1.682–5.242)	<0.001	0.765 (0.255–2.298)	0.633
Malignancy	22 (37.9)	1.823 (1.065–3.123)	0.029	0.964 (0.525–1.769)	0.905
IBD	0	NA	NA	NA	NA
CVA	11 (19.0)	1.917 (0.982–3.741)	0.056	NA	NA
HSCT	8 (13.8)	3.930 (1.813–8.520)	0.001	0.890 (0.325–2.440)	0.821
Median CCI score (IQR)	4 (2–6)	1.360 (1.233–1.498)	0.001	1.223 (0.775–1.931)	0.387
No. days before index patient treatment, median (IQR)	1 (0–2)	1.049 (0.861–1.277)	0.636	NA	NA
Laboratory test					
Leukocyte count >15,000/uL	12 (20.7)	2.236 (1.164–4.295)	0.016	1.132 (0.589–2.173)	0.710
Median CRP, mg/L (IQR)	42.9 (16.3–82.7)	1.005 (1.003–1.007)	<0.001	1.002 (0.999–1.004)	0.209
Mean albumin, g/dL (SD)	3.02 (0.52)	1.671 (0.572–4.881)	0.347	NA	NA
Clinical conditions					
Ileostomy	1 (1.7)	1.520 (0.203–11.366)	0.683	NA	NA
Enteral tube insertion	23 (39.7)	4.718 (2.750–8.095)	<0.001	1.432 (0.826–2.484)	0.201
Contact cases§	126	NA	NA	NA	NA
Group 1¶	44 (34.9)	2.065 (1.199–3.559)	0.009	1.125 (0.789–1.604)	0.516
Room sharing#	11 (25.0)	0.566 (0.129–2.474)	0.449	NA	NA
Contact during diarrhea episode**	13 (29.5)	0.313 (0.072–1.363)	0.122	NA	NA
Group 2††	11 (8.7)	0.605 (0.240–1.525)	0.287	NA	NA
Group 3‡‡	71 (56.4)	0.664 (0.393–1.121)	0.126	NA	NA
Median contact time, min (IQR)	4,320.0 (128.8–10,080.0)	1.0	0.849	NA	NA
Deaths	11 (19.0)	3.570 (1.814–7.023)	<0.001	NA	NA

*Values are no. (%) except as indicated. aOR, adjusted odds ratio; CCI, Charlson comorbidity index; CDI, *Clostridioides difficile* infection; CHF, chronic heart failure; CKD, chronic kidney disease; COPD, chronic obstructive pulmonary disease; CRP, C-reactive protein; CVA, cerebrovascular accident; DM, diabetes mellitus; HSCT, hematopoietic stem cell transplantation; HTN, hypertension; IBD, inflammatory bowel disease; NA, not applicable; OR, odds ratio.
†A generalized linear model was used to calculate the odds of subsequent CDI occurrence in the univariate analysis.
‡A generalized linear mixed model adjusted for all variables exhibiting statistical significance in the univariate analysis was used to calculate the odds of subsequent CDI occurrence.
§Because 744 patients experienced >2 episodes of contact from separate index patients, a disparity emerged between the number of contact cases and the number of contact patients.
¶Group 1 included patients who had direct contact with index patients.
#Co-hospitalization in the same bedroom with the index patient for a period >24 hours.
**Contact history during the manifestation of diarrhea of the index patient.
††Group 2 included patients who had indirect contact with index patients via healthcare personnel.
‡‡Group 3 included patients who had indirect contact with index patients via the environment.

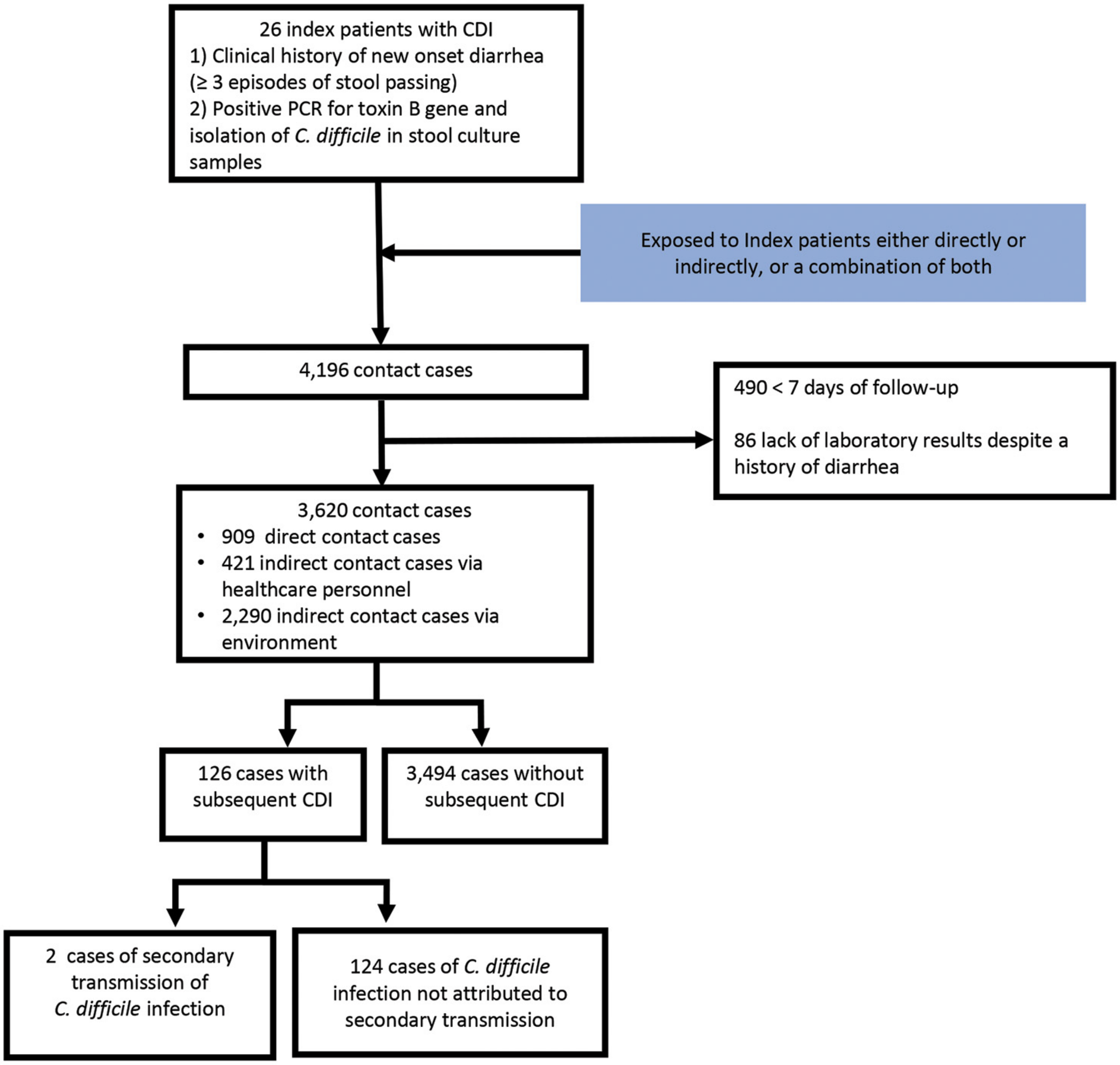

Appendix 1 Figure 1. Flowchart depicting the contact cases of index patients with *Clostridioides difficile* infection included in study identifying contact time required for secondary transmission of *C. difficile* infections. Real-time locating system was used to track contact cases. Box on right indicates the exclusion criteria for study participants and number of cases excluded. CDI, *Clostridioides difficile* infection.

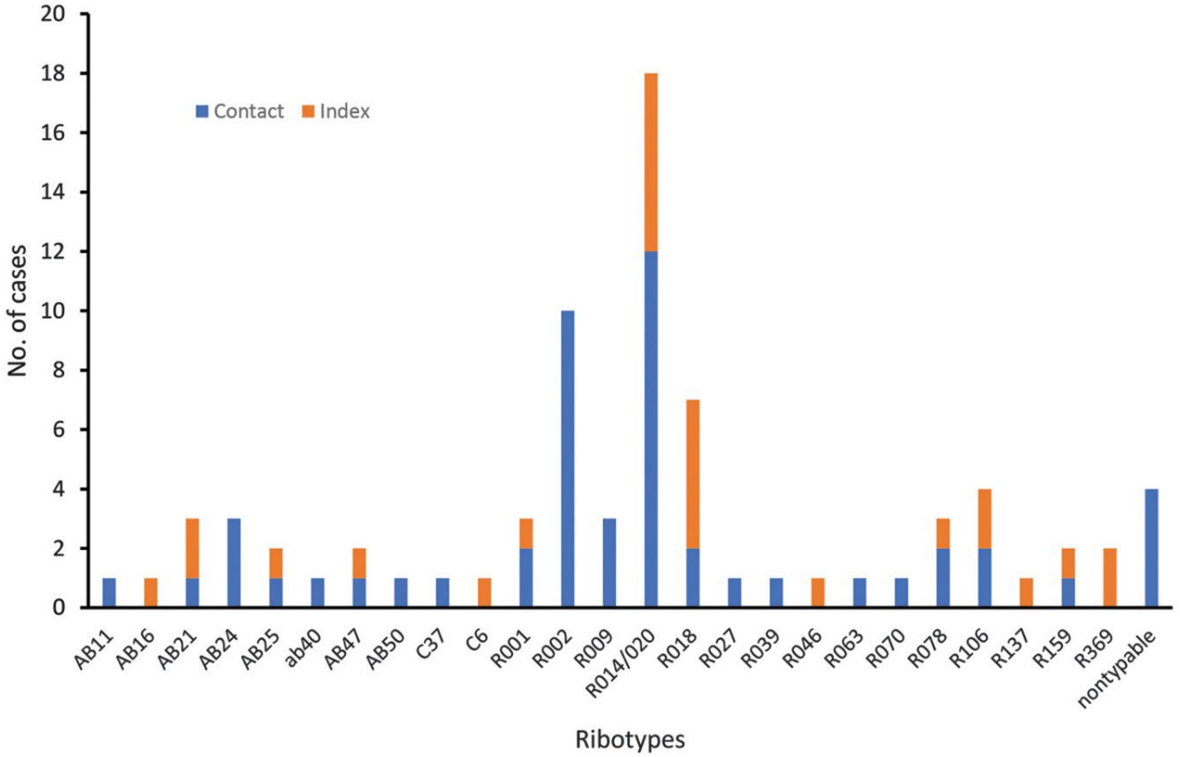

Appendix 1 Figure 2. Distribution of different *Clostridioides difficile* ribotypes identified in index and contact cases. Study identified contact time required for secondary transmission of *C. difficile* infections by using real-time locating system. Nontypable indicates the strain was unidentifiable by using PCR ribotyping.

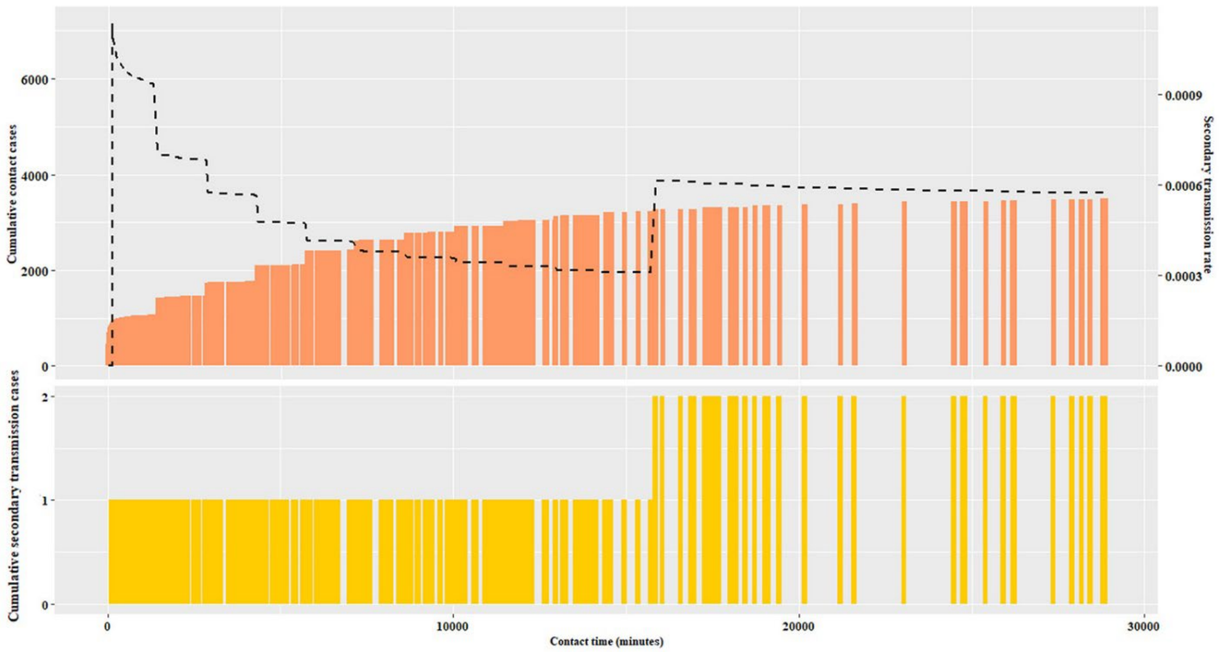

Appendix 1 Figure 3. Cumulative secondary transmission rate of *Clostridioides difficile* infection in relation to contact time. Colored bars indicate the cumulative occurrence of contact cases (upper panel) and secondary transmission (lower panel). Dotted line represents the cumulative secondary transmission rate at the designated contact time. Cumulative secondary transmission rate was calculated by dividing the cumulative number of secondary transmission cases by the cumulative number of contact cases.
